# The effect of post-fixation olecranon lengthening on range of motion of the elbow: a cadaveric study

**DOI:** 10.1186/s12891-021-04936-9

**Published:** 2021-12-20

**Authors:** Khai Phang Wong, James Chung Hui Tan

**Affiliations:** Department of Orthopaedic Surgery, Khoo Teck Puat Hospital, 90 Yishun Central, Singapore, 768828 Singapore

**Keywords:** olecranon plating, olecranon lengthening, comminuted fractures, elbow extension, osteotomy, cadaveric study

## Abstract

**Background:**

Principles of fixation of comminuted olecranon fractures include anatomical reduction of the articular surface and restoration of ulnohumeral joint motion. However, comminution sometimes may not permit anatomical fixation of fracture fragments, resulting in inadvertent olecranon lengthening after plate fixation. The aim of our study is to investigate the relationship between olecranon lengthening following plate fixation and loss of elbow extension.

**Materials and methods:**

Transverse olecranon osteotomies were performed on 8 cadaveric elbows. The osteotomy sites were then fixed with olecranon plates. Lengthening of the osteotomy sites were simulated by placement of 2mm, 4mm, 6mm and 8mm blocks. Lateral view photographs of the elbows were taken after each degree of lengthening. These photographs were then printed and measurements of elbow extension were performed with a goniometer with average values taken. The measurements were tabulated and statistical analysis performed to determine the relationship between degree of elbow extension loss and amount of olecranon lengthening.

**Results:**

Average values of each degree of lengthening (at 2mm, 4mm, 6mm and 8mm) were taken and compared with the baseline measurement (at 0mm). Cluster analysis showed that for every increment in osteotomy length of 2mm, there is a corresponding increase of 0.79° of elbow extension loss (p<0.01, 95% confidence level 0.55°-1.03°).

**Conclusion:**

Lengthening of olecranon by increments of 2mm correlates positively with loss of elbow extension. This shows that inadvertent intra-operative olecranon lengthening post-fixation may result in limited range of motion. However, it is reassuring to know that the small degree of extension loss may not translate to functional limitation.

## Background

Olecranon fractures constitute about 10% of all upper extremity fractures [1]. These fractures usually occur around the fifth decade of life and frequency increases with age reaching a peak during the seventh decade [2]. There is equal gender distribution, with a similar age-related increase [2]. Various classification systems have been described, with the Mayo classification most commonly used by surgeons as it provides a guide to treatment. It is based on displacement, stability and comminution of the fracture fragments [3]. Type II and III injuries usually require surgical treatment.

Plate fixation is the preferred method of surgical fixation in fractures with comminution and bone loss, providing a stable construct with a low rate of hardware removal and allowing early functional rehabilitation with good union rates and clinical outcomes [4–9].

Loss of elbow extension is one of the most commonly reported complications of olecranon plating [5, 6, 9–15], potentially leading to poor functional outcomes. Extension deficit can be attributed to various causes. We believe that inadvertent olecranon lengthening during surgery is an under-reported cause.

The aim of this study is to determine the relationship between olecranon lengthening and loss of elbow extension. We hypothesize that olecranon lengthening leads to limitation of full elbow extension and potentially results in adverse outcomes. To test this hypothesis, a cadaveric study was conducted.

## Materials and methods

### Specimens

8 fresh cadaveric elbows (4 males and 4 females, mean age 74 years, with a range from 61 to 98 years) were obtained from Science Care Inc, Phoenix, Arizona, United States of America. The use of human cadaveric specimens for this study was approved by the local ethics committee. All cadaveric elbows did not have pre-existing deformities or prior dissections. The specimens were kept in a storage fridge at a standard temperature. The individual specimens were then taken out for thawing one day prior to the conduct of the experiment.

### Measurement landmarks

**Fig. 1:**
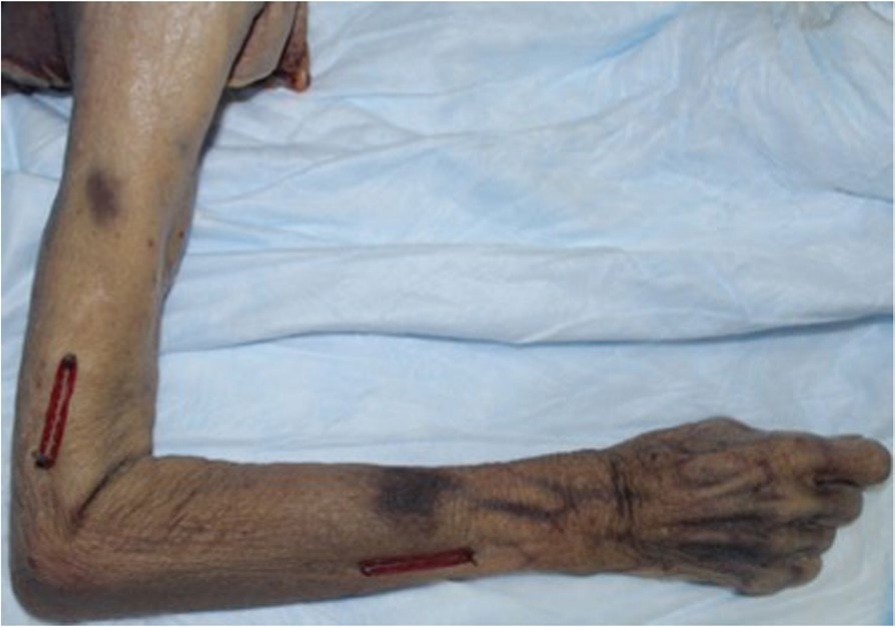
Drill bits inserted 5 and 10 cm from the tip of the olecranon and 5 and 10 cm from the ulna styloid as surrogate for measurements of longitudinal axes of the humerus and forearm respectively

Drill bits were inserted into the humerus and the ulna at standardized locations for measurements (5cm and 10cm from the tip of the olecranon, and 5cm and 10cm from the ulna styloid respectively). These were then tagged with red rubber bands as surrogates for the axes of the humerus and forearm (Fig. 1).

### Approach

**Fig. 2:**
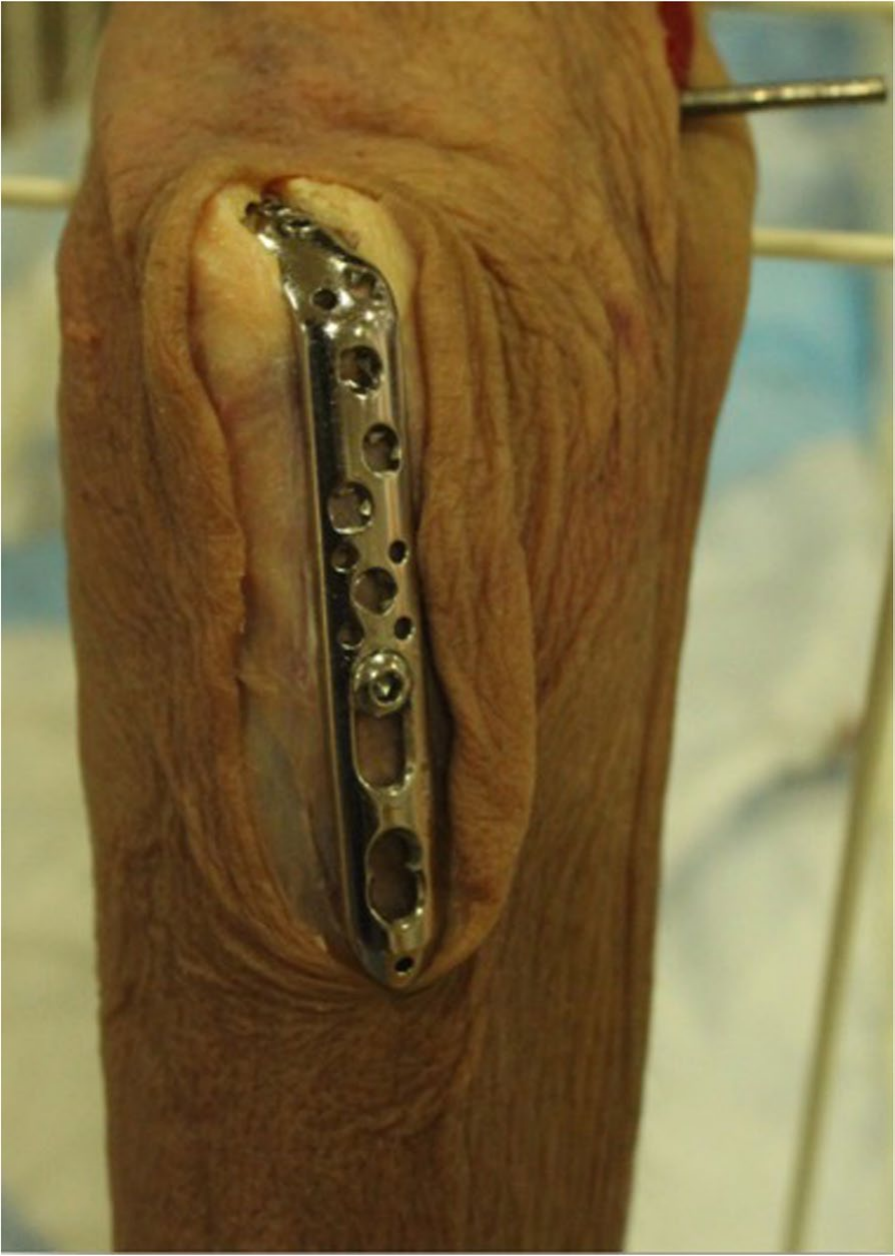
The proximal ulna is exposed and fixed with a proximal olecranon plate

A standard posterior midline incision was used, starting proximal to the tip of the olecranon extending to the proximal aspect of the ulna. The triceps tendon was split at its insertion to accommodate the plate. The proximal ulna was then exposed. The specimens were then fixed with a 2.7/3.5 VA-LCP proximal olecranon plate (Synthes, Oberdorf, Switzerland) (Fig. 2). Two 2.7mm locking screws were used to fix the proximal fragment and one 3.5mm cortical screw was used to secure the plate to the ulna shaft.

### Osteotomy

**Fig. 3:**
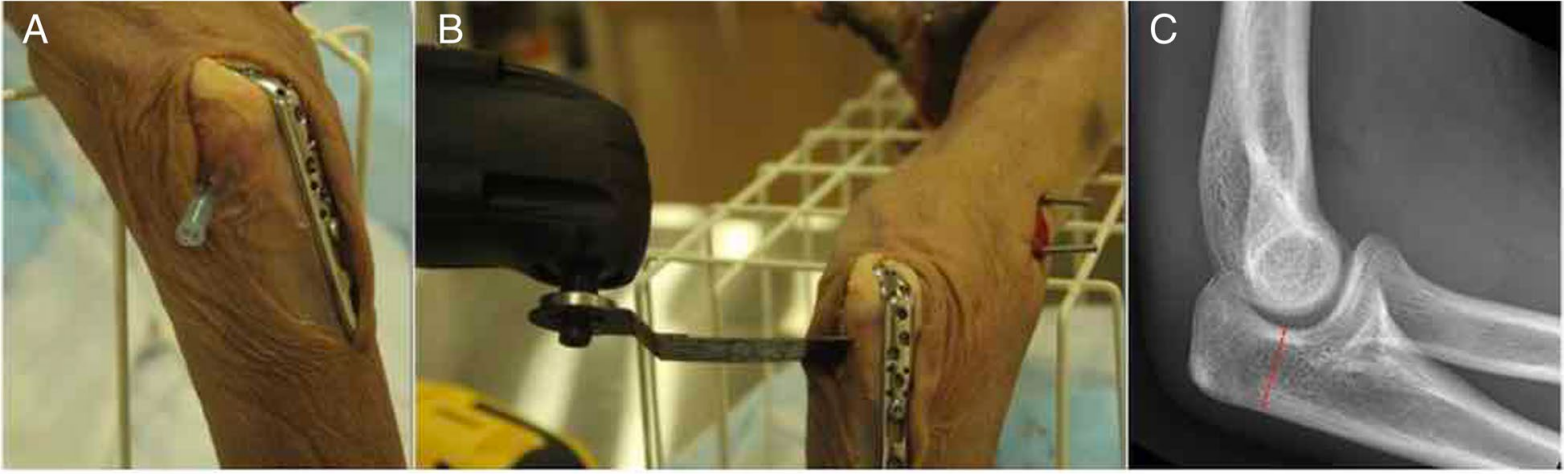
Lowest point of trochlear is marked with needle (**A**). Osteotomy is performed at the marked point (**B**). Radiograph demonstrating location of osteotomy (**C**)

A transverse osteotomy is made at the lowest point of the trochlear notch, which is identified with the aid of a needle (Fig. 3). A small incision was made to expose the osteotomy site, taking care to preserve as much capsule and adjacent ligaments as possible.

### Measurements

**Fig. 4:**
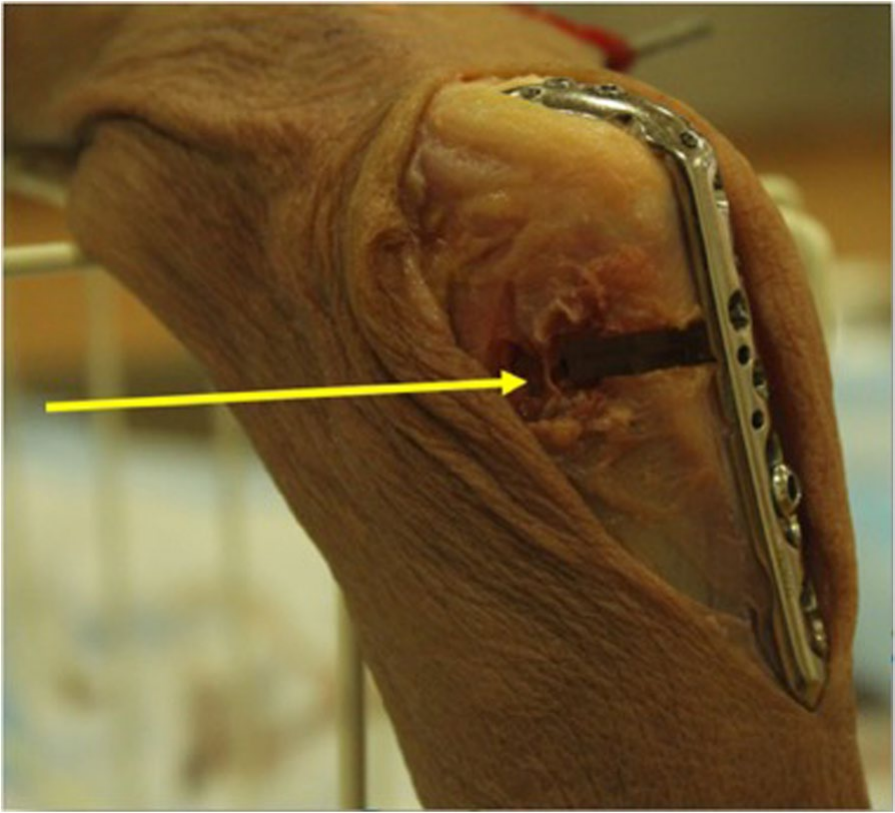
Olecranon lengthening simulated by placing blocks of varying thickness into the osteotomy site

**Fig. 5:**
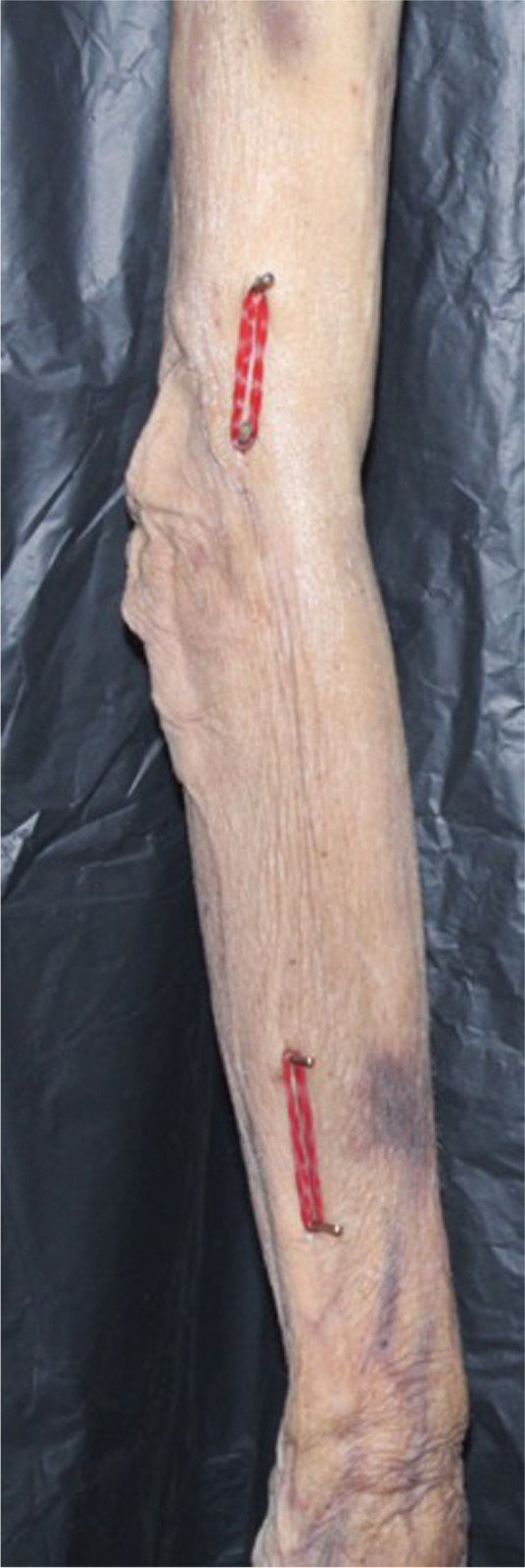
The cadaveric elbows were positioned with the humerus vertical the elbow extended by the pull of gravity. Photographs were taken at baseline (0mm) and at interval increments of 2mm

Olecranon lengthening was simulated by placing blocks of varying thickness (2mm, 4mm, 6mm and 8mm) into the osteotomy site (Fig. 4). The cadaveric elbows were positioned such that the humerus was vertical and the elbow was extended by the pull of gravity. Photographs were taken at baseline (0mm) and at interval increments of 2mm (Fig. 5). The measurement at 0mm was taken with the plate fixed and osteotomy performed, but without any blocks inserted. The photographs were then printed and measurements were done using a goniometer. To improve measurement accuracy, multiple photographs were taken at each increment and an average of the measurements was obtained. Equipment used was standardized for all 8 cadaveric specimens.

### Data analysis

The average measurements for elbow extension (in degrees) at 2mm, 4mm, 6mm and 8mm were obtained for each cadaver and subtracted from the baseline (0mm). Cluster analysis was performed with Stata IC Version 13.1 (StataCorp, Texas, U.S.) using the linear mixed effects model. The data was entered into tables and analysis was performed with the commands “xtmixed angle” (representing degree of extension deficit) and “osteo” (representing amount of osteotomy). Data was also plotted on graph using Microsoft Excel (Microsoft, Washington, U.S.A.).

## Results

Table 1 shows the various degrees of extension deficits for various osteotomy lengths in each of the 8 cadavers. As we had 8 different cadavers each with different sets of data, we used the linear mixed effects model to allow for clustering of data, with each cadaver analysed as a cluster.

**Table 1:** Table showing degrees of elbow extension deficits at various osteotomy lengths in 8 cadaveric elbows

Osteotomy length (mm)	Extension deficit (degrees)							
	Cadaver 1	Cadaver 2	Cadaver 3	Cadaver 4	Cadaver 5	Cadaver 6	Cadaver 7	Cadaver 8
2	0.25	4.25	1.25	2.00	2.50	0.00	1.75	0.00
4	2.25	4.50	1.25	2.25	5.00	6.00	2.25	5.50
6	2.50	5.00	2.75	2.50	6.25	7.00	3.50	6.25
8	5.75	7.50	3.25	2.75	6.75	8.00	10.25	8.00

**Fig. 6:**
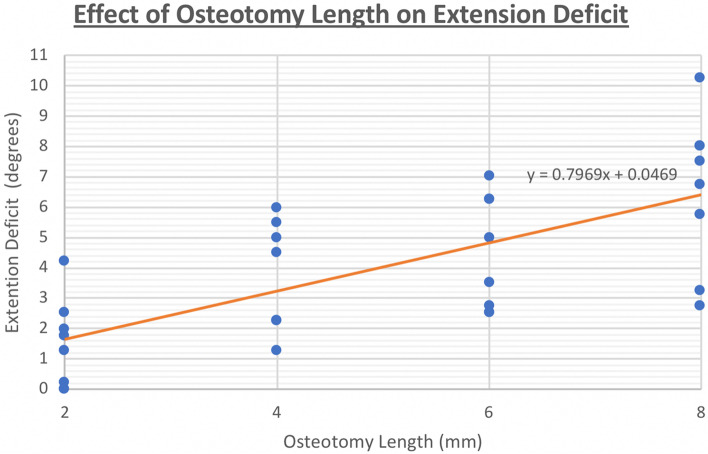
Linear regression analysis of relationship between osteotomy length (in mm) and elbow extension deficit (in degrees)

We found that in each of the cadavers, an increment in the osteotomy length generally results in an increase in the amount of extension deficit. Cluster analysis showed that for every increment in osteotomy length of 2mm, there is a corresponding increase of 0.79° of elbow extension loss, with a p value of >0.01 and a 95% confidence level from 0.55° to 1.03°. Fig. 6 shows a graphic representation of the linear regression relationship between osteotomy length and elbow extension deficit.

## Discussion

The goals of surgical treatment in olecranon fractures are to restore articular congruity and joint stability for optimal bone healing, so as to allow early mobilization to achieve a pain-free functional elbow with good range of motion. Loss of elbow function can cause significant disability and adversely affect daily activities, work and recreational activities.

Tension band wiring has been shown to provide stable fixation with a good union rate in non-comminuted olecranon fractures with transverse configurations [16]. However, tension-band constructs in comminuted olecranon fractures may cause failure in compression due to subchondral bone comminution [4]. This causes the fracture fragments to collapse, leading to a narrowed surface for olecranon articulation and poor tracking [17]. In a mechanical study conducted by Fyfe et al, while tension-band wiring afforded adequate rigidity in models with transverse osteotomies, a significantly more stable fixation was achieved by plate fixation in more comminuted fractures [18]. Also, initiating early movement after tension band wiring may cause problems in comminuted fractures with bone loss [5]. Plate fixation has become standard treatment for comminuted olecranon fractures, and various clinical and biomechanical studies have reported favourable results, good functional scores and satisfactory range of motion [4–9, 15].

The popularization of posterior plating in comminuted olecranon fractures has also led to extensive research about its adverse consequences. The most common reported complication following plate fixation of comminuted fractures is loss of elbow extension [5, 6, 9–15]. Various studies have reported extension deficits ranging from 9.5° [10] to greater than 30° [6, 12].

In 2016, De Giacomo et al reported that the most common deficiency in patients after olecranon plating was lack of full elbow extension [14]. In his study of 163 patients, 39% of patients lacked at least 10° of extension compared to the contralateral limb, with a higher incidence in open fractures, comminuted fractures and fractures with diaphyseal extension. These patients were also found to have higher Disabilities of the Arm, Shoulder and Hand (DASH) scores (i.e. more severe disability) than their counterparts who achieved full elbow extension.

The exact etiology behind loss of terminal elbow extension after olecranon plate fixation has been poorly described in the literature. Hak and Golladay [19] found that loss of elbow motion was worse in patients with concomitant fractures of the radial head, capitellum, coronoid or Monteggia fracture-dislocations. Anderson et al [5] postulated that loss of elbow extension was due to the proximal aspect of the plate sitting in close proximity to the triceps mechanism. However, it is evident that extension deficits still persist even after modernization of the olecranon plate allowing more anatomical fit to the proximal ulna.

The proximal ulna comprises the coronoid and the olecranon process, making up the saddle-shaped ellipsoid articular surface of the sigmoid notch [20]. The olecranon process plays an integral role in maintaining passive stability of the elbow joint as serial resection of the olecranon results in a linear reduction in stability [20, 21].

The midpoint from the coronoid process to the tip of the olecranon is covered by a transverse area devoid of cartilage [22]. Over-compression of this area during fracture reduction is undesirable as it may lead to a narrowed trochlear fossa and an incongruent radius of curvature [17]. We believe the converse is also true in the case of lengthening.

Normal elbow range of motion is from 0° to 150° [20]. Morrey et al demonstrated that functional elbow range of motion ranges from 30° to 130° [23]. Fischer et al showed that elbow flexion and extension occurred around a centre of rotation involving an area of 2 to 3 mm in diameter at the trochlear [23]. During elbow extension, the olecranon fossa accommodates the olecranon process of the ulna. This articulation is lost in displaced fractures and also in comminuted fractures that are plated incorrectly resulting in advertent olecranon lengthening. This may ultimately result in limitation to full elbow extension.

Bailey et al in 2001 [4] emphasized the importance of restoring the normal anatomy of the trochlear notch, in particular the anterior and posterior facets of the olecranon. They also mentioned in their study that severe comminution may result in a gap between the facets and olecranon shortening does not affect the outcome as long as the contour of the olecranon notch is maintained. However, this has not been substantiated in their study. Some authors argue that loss of terminal flexion and extension are usually minor and functionally insignificant [16], but the degree of which were not specified. In 1987, Murphy et al [24] studied olecranon fractures and concluded that an articular displacement or mal-reduction greater or equal to 2mm was associated with poorer results in terms of pain and range of motion. To our best knowledge, there have been very few studies in the current literature investigating the effects of olecranon lengthening following suboptimal olecranon fixation.

Our present study shows that lengthening of the olecranon at the articular surface has a positive correlation to loss of terminal elbow extension after fixation with an anatomical olecranon plate. We believe that in patients with severely comminuted fractures, improper plate placement or non-anatomic fracture reduction may result in inadvertent olecranon lengthening. If this lengthening is not recognized intraoperatively, this may result in post-operative limitation of terminal elbow extension. However, due to the relatively small degree of extension loss of 0.79°for every 2mm lengthening, it may be reassuring to know that this may not translate to functional limitation. We also note that in vivo, post-operative immobilization and rehabilitation protocols play important roles in determining extension loss, which we were unable to determine using our cadaveric study.

We recognize a few limitations of our present study. As with most cadaveric studies, other important factors that influence elbow movement such as blood supply, triceps and soft tissue integrity and healing response of the bone are not taken into account, and may be confounding factors. Many of the specimens are intrinsically contracted, which may affect degree of lengthening. We established a baseline measurement after performing the osteotomy and applying the plate without any extension blocks being placed, and took reference from this baseline measurement to compare with the subsequent ones after simulated lengthening.

We were also limited by a small sample size of 8 cadaveric elbows with no control group. Therefore, we used a linear mixed effects model to allow for clustering of data, with each cadaver analysed as a cluster. As standard error is small, cluster analysis makes our data achieve statistical significance.

A potential method to improve our result accuracy is to incorporate the use of fluoroscopy. Wadia et al [25] stated that the trochlear width as seen on the lateral view is the line that will be most affected in olecranon fractures and is the single most important parameter to be reconstructed. He studied 100 normal adult antero-posterior and lateral elbow radiographs and derived 3 measurement ratios to help determine a more accurate and reliable method to measure intra-operative olecranon length. Incorporating these measurements in our study would allow us to better quantify olecranon lengthening. We also advocate use of these fluoroscopic parameters intra-operatively to ensure adequate restoration of olecranon length, especially in cases of severe comminution.

## Conclusion

In conclusion, our cadaveric study shows a significant association between olecranon lengthening in 2mm increments up to 8mm and loss of elbow extension. We believe that inadvertent olecranon lengthening following plate fixation is an under-reported cause of elbow extension deficit post-surgery. This usually results from non-anatomic reduction of fracture fragments in cases with severe comminution and bone loss. We advocate use of radiological parameters intraoperatively to ensure adequate restoration of olecranon length.

## Data Availability

The datasets generated and/or analysed during the current study are not publicly available due to patient confidentiality but are available from the corresponding author on reasonable request.
